# Pregnancy-related healthcare utilisation in Agincourt, South Africa, 1993–2018: a longitudinal surveillance study of rural mothers

**DOI:** 10.1136/bmjgh-2021-006915

**Published:** 2021-10-07

**Authors:** Daniel E Sack, Ryan G Wagner, Daniel Ohene-Kwofie, Chodziwadziwa W Kabudula, Jessica Price, Carren Ginsburg, Carolyn M Audet

**Affiliations:** 1 Vanderbilt Institute of Global Health, Vanderbilt University Medical Center, Nashville, Tennessee, USA; 2 Medical Research Council/Wits Rural Public Health and Health Transitions Research Unit (Agincourt), School of Public Health, Faculty of Health Sciences, University of the Witwatersrand, Johannesburg, South Africa

**Keywords:** maternal health, health systems, obstetrics, epidemiology

## Abstract

**Introduction:**

Pregnancy-related health services, an important mediator of global health priorities, require robust health infrastructure. We described pregnancy-related healthcare utilisation among rural South African women from 1993 to 2018, a period of social, political and economic transition.

**Methods:**

We included participants enrolled in the Agincourt Health and Socio-Demographic Surveillance System in Mpumalanga Province, South Africa, a population-based longitudinal cohort, who reported pregnancy between 1993 and 2018. We assessed age, antenatal visits, years of education, pregnancy intention, nationality, residency status, previous pregnancies, prepregnancy and postpregnancy contraceptive use, and student status over the study period and modelled predictors of antenatal care utilisation (ordinal), skilled birth attendant presence (logistic) and delivery at a health facility (logistic).

**Results:**

Between 1993 and 2018, 51 355 pregnancies occurred. Median antenatal visits, skilled birth attendant presence and healthcare facility deliveries increased over time. Delivery in 2018 vs 2004 was associated with an increased likelihood of ≥1 additional antenatal visits (adjusted OR (aOR) 10.81, 95% CI 9.99 to 11.71), skilled birth attendant presence (aOR 4.58, 95% CI 3.70 to 5.67) and delivery at a health facility (aOR 3.78, 95% CI 3.15 to 4.54). Women of Mozambican origin were less likely to deliver with a skilled birth attendant (aOR 0.42, 95% CI 0.39 to 0.45) or at a health facility (aOR 0.43, 95% CI 0.41 to 0.46) versus South Africans. Temporary migrants reported fewer antenatal visits (aOR 0.35, 95% CI 0.33 to 0.38) but were more likely to deliver with a skilled birth attendant (aOR 1.91, 95% CI 1.66 to 2.2) or at a health facility (aOR 1.4, 95% CI 1.24 to 1.58) versus permanent residents.

**Conclusion:**

Pregnancy-related healthcare utilisation and skilled birth attendant presence at delivery have increased steadily since 1993 in rural northeastern South Africa, aligning with health policy changes enacted during this time. However, mothers of Mozambican descent are still less likely to use free care, which requires further study and policy interventions.

**Key questions**

**What is already known?**

- Adequate healthcare during the antepartum, peripartum and postpartum periods require an accessible health system with the infrastructure and personnel to meet the unique healthcare needs of individuals during and after pregnancy.
- Number of antenatal visits, skilled birth attendant presence at delivery and delivery at a health facility are all proxies for a functional pregnancy-related health system.
- Improving pregnancy-related care is a global health priority.

**What are the new findings?**

- In this observational study using surveillance data, we found that antenatal visits, skilled birth attendant presence and health facility delivery increased over time among women living in rural northeastern, South Africa.
- Nationality and residency status were meaningful predictors of having a skilled birth attendant present at delivery and delivering in a health facility, both of which also increased over time.

**What do the new findings imply?**

- Despite major improvements in pregnancy-related healthcare utilisation, mothers of Mozambique origin in northeastern South Africa are still less likely to have a skilled birth attendant at delivery or deliver at a health facility, which requires attention.
- Mothers who identify as temporary migrants have different pregnancy-realted healthcare utilisation patterns than mothers who identify as permanent residents, which suggests policy interventions will likely need to be tailored to residency status in this population.

## Introduction

Adequate pregnancy-related care requires an accessible health system with the infrastructure and personnel to meet the unique healthcare needs of women during the antepartum, peripartum and postpartum periods. Women must be able to safely travel to receive antenatal care and give birth in the presence of a skilled birth attendant.^1–4^ South Africa’s most recent strategic plan to improve maternal and child health (2015) and guidelines for maternity care (2016) target a number of maternal and child indicators.^5 6^ These include increasing postpartum contraceptive uptake, a minimum of four antenatal visits per pregnancy and increasing the proportion of births attended by skilled birth personnel through improved access to health facility deliveries.^5 6^

In the last 30 years, South Africa has undergone rapid sociopolitical changes, including the end of apartheid and the rise of the HIV epidemic.^7 8^ These changes have had a profound impact on healthcare delivery, including pregnancy-related care.^7 8^ The establishment of South Africa’s first freely elected government after apartheid in 1994 brought sweeping changes. There was a renewed focus on health and development in formerly disenfranchised, generally rural areas and more centralised healthcare delivery coordinated through the national department of health.^7–11^

The HIV pandemic has also impacted all aspects of South Africa’s health system, including pregnancy-related healthcare.^6 12–14^ Although HIV first appeared in South Africa in the 1980s, HIV prevalence ballooned from 1% in 1991 to 16% in 2000 in the general population and from 0.7% in 1990 to 22.4% in 1998 among women attending antenatal visits (although antenatal testing only became widely available in 2005).^15–17^ Despite this, some government officials promoted ‘natural remedies’,^7 15 16 18–20^ delaying the start of South Africa’s national antiretroviral campaign until 2004.^15 21^ HIV testing and treatment during and after pregnancy has since been implemented to reduce maternal and infant morbidity and mortality and prevent mother-to-child HIV transmission.^5 14^

Through a combination of postapartheid and HIV-induced health system changes, as recently as 2016, approximately 76% of women attended at least four antenatal care visits, 97% of births were attended by a skilled provider, and 96% of deliveries were in a health facility—an increase from 74%, 84% and 83%, respectively, in 1998.^22 23^ This analysis further interrogates these improvements with data from the Agincourt Health and Socio-Demographic Surveillance System (HDSS) in the Bushbuckridge subdistrict.^9 10^ We first describe indicators of pregnancy-related healthcare use among women who were part of the surveillance population and report on a number of sociodemographic, health access, utilisation and delivery indicators and outcomes from from 1993 to 2018. We then characterise predictors of antenatal care utilisation, presence of a skilled birth attendant at delivery, and delivery in a health facility to explore the factors contributing to pregnancy-related healthcare utilisation in this population.

## Methods

### The Agincourt HDSS

The Agincourt HDSS, part of the Bushbuckridge subdistrict in Mpumalanga Province, northeast South Africa, was established in 1992 to support local health system development in the postapartheid era.^9 10^ Since 1992, the Agincourt HDSS team has conducted annual surveys with a population that has grown from 8900 households in 1992, to over 22 000 households in 2018.^9 10^ In 2018, the Agincourt HDSS covered roughly 120 000 people residing in 31 villages.^9 10^ Roughly 30% of the population arrived as refugees from Mozambique during the Mozambican civil war.^9 10^ As part of annual Agincourt HDSS census updates, where field staff collect demographic and household data of individuals in surveillance system, they collect full pregnancy and maternal histories of women who became pregnant or gave birth during the previous year and documented births missed from previous years.^9 10^

### Data preparation

**Figure 1:**
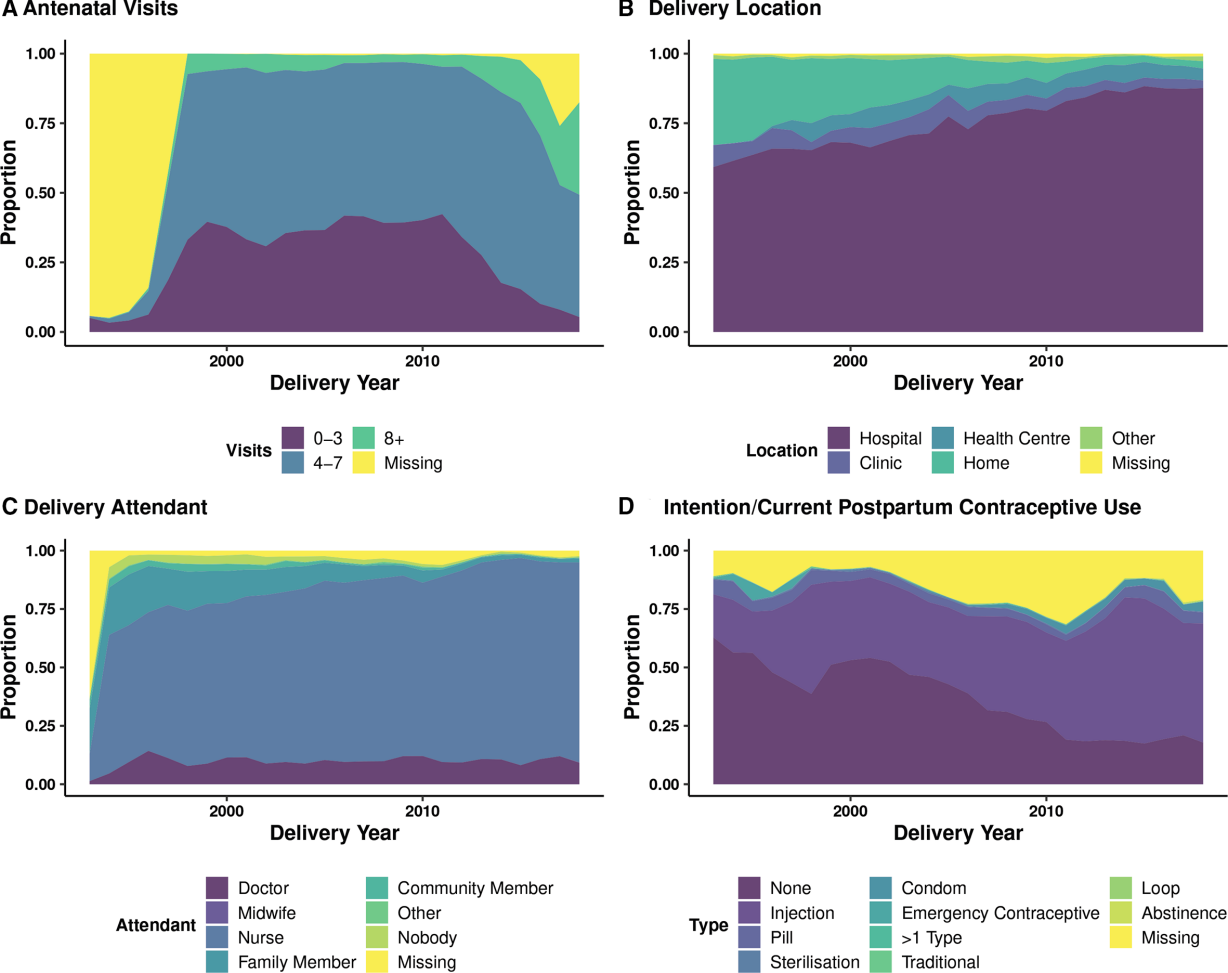
Pregnancy-related health utilisation by delivery year. All subfigures present the the proportion of pregnancies per year. (A) Shows antenatal visits by delivery year, with number of visits categorised into four bins: 0–4, 5–8, 9+ and missing. (B) Shows delivery location by delivery year. (C) Shows the reported delivery attendant by delivery year. (D) Shows postpartum pregnancy intention or use by delivery year.

**Figure 2:**
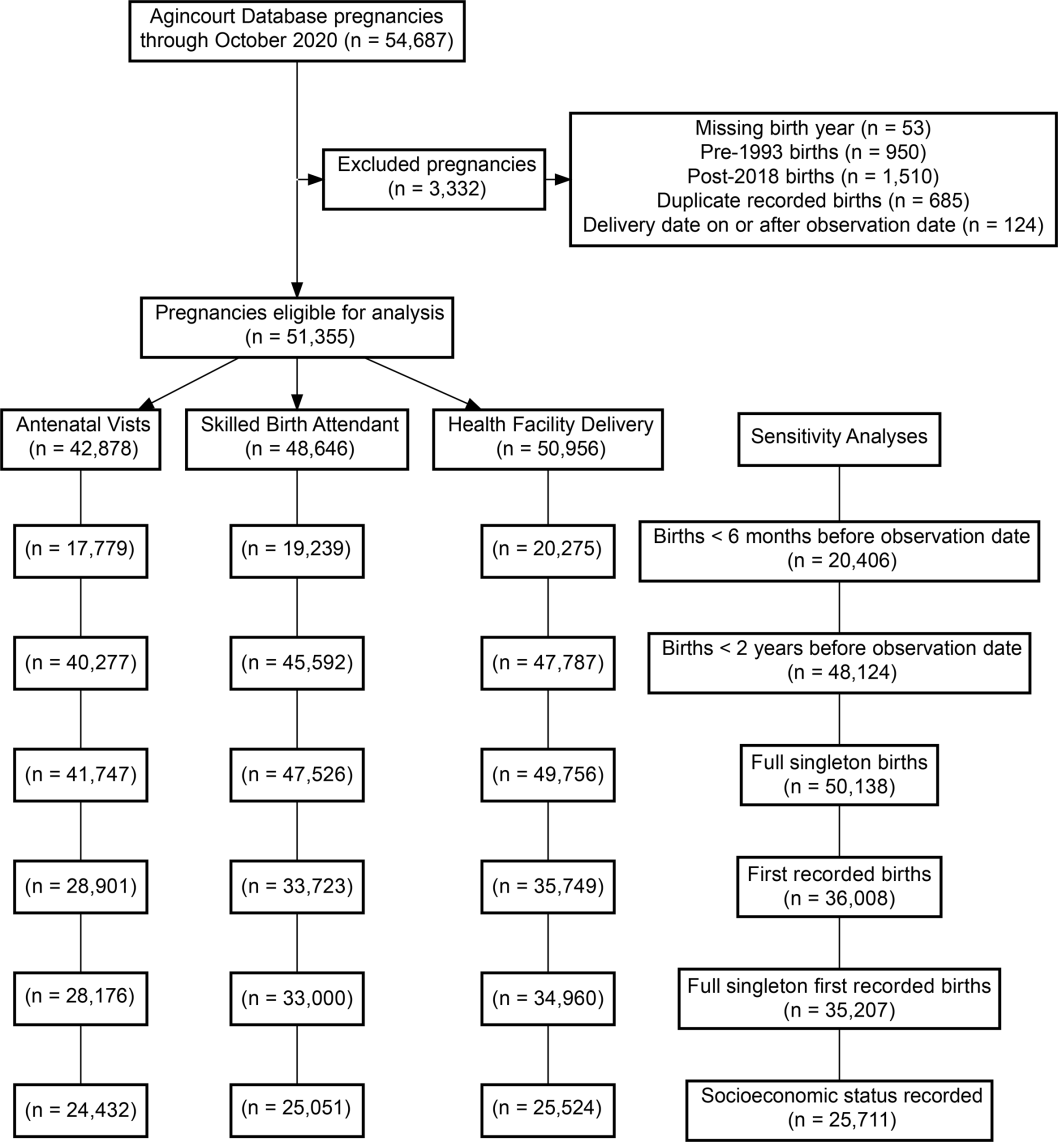
Study flow chart presents participant eligibility for each step of the analysis.

This analysis included cross-sectional surveillance data from women aged 11–55 whose births from 1993 to 2018 were recorded in the Agincourt HDSS database (n=51 355, table 1, figure 1, online supplemental efigure 1). We excluded pregnacies recorded for the same women in the same year with the same birth outcome (presumed duplicate records) between 1993 and 2018 and births that took place outside the study period (including pregnancies with missing delivery dates) (n=3332) (figure 2). For each predictive model, we included pregnancies with non-missing outcomes (antenatal visits, skilled birth attendant presence and delivery location) (figure 2). Age at delivery was calculated from the mother’s date of birth and the date of delivery. Calculated ages less than 11 and greater than 55 were assumed to be secondary to data entry errors and marked as missing. We imputed zero antenatal visits to the 610 cases where the number of antenatal visits were missing, but the dichotomised value for having attended an antenatal clinic was ‘No’ in an auxillary Agincourt HDSS variable for all analyses. Reported antenatal visits values greater than 15 were assumed to be data error errors and coded as missing. We coded education as a numerical value, which allowed us to standardise years of reported South African and Mozambican school, as well as adult educational programmes such as the adult basic education and training and national qualification framework in South Africa to their equivalent years of education. The proportion of women using contraception before and after pregnancy was presented by method in table 1 and dichotomised to use modern contraceptive methods or not (modern contraceptives included: condoms, emergency contraceptives, injectables, loops, more than one contraceptive, long-acting reversible contraceptives, pills and sterilisation) in the multivariable models. Pregnancy intention was coded as intended, unintended or not reported, and student status was dichotomous (yes vs no), both were unchanged from the AHDSS dataset. Like in the Agincourt HDSS dataset, nationality was coded as South African, Mozambican (combined pre-1992 and post-1992 arrival) and other (any other country) and migrant status was coded as resident (lived more than 6 months in the area over the previous year), temporary migrant (lived fewer than 6 months in the area due to work) and other migrant (in the study area for education, to care for a family member, or another reason and did not plan to stay permanently). Time between delivery and observation date was calculated as days between the delivery date and the observation date for sensitivity analyses. While an asset-based household socioeconomic status variable is available in the Agincourt HDSS,^24^ it has only been calculated every other year since 2001 and every year from 2014 onwards, so there was too much missing data for it to be included in the primary analysis. It was, however, included in a sensitivity analysis as a linear continuous variable (see below). Detailed information on variable coding and variables included in each stage of the analysis is available in online supplemental etables 1–4. Missing data are characterised in online supplemental etables 5.

10.1136/bmjgh-2021-006915.supp1 Supplementary data

**Table 1:** Descriptive data by delivery year (5 years increments, after 1999)

n (%) or median (IQR)	Delivery year				
	1993–1998*	1999–2003	2004–2008	2009–2013	2014–2018
Pregnancies	10 407	8345	9518	11 483	11 602
First time pregnancies	8651 (83.1)	5368 (64.3)	6104 (64.1)	8039 (70)	7846 (67.6)
Fertility rate	2.9 (2.9–3.2)	2.8 (2.5–2.9)	2.7 (2.6–2.8)	2.5 (2.4–2.5)	2.0 (1.9–2.2)
Median age	25.1 (20–31.5)	24.9 (19.8–31.2)	24.6 (19.8–30.6)	24.9 (20.4–30.7)	25.8 (21.2–31.3)
Missing	47 (0.5)	0 (0)	2 (0)	1 (0)	6 (0.1)
Median antenatal visits	4 (2–6)	4 (3–5)	4 (2–5)	4 (2–5)	6 (5–7)
0–3	1206 (11.6)	2967 (35.6)	3732 (39.2)	4181 (36.4)	1369 (11.8)
4–7	1825 (17.5)	4883 (58.5)	5379 (56.5)	6710 (58.4)	6728 (58)
8+	217 (2.1)	484 (5.8)	364 (3.8)	535 (4.7)	2298 (19.8)
Missing	7159 (68.8)	11 (0.1)	43 (0.5)	57 (0.5)	1207 (10.4)
Median years of education	10 (10–11)	11 (10–11)	11 (10–11)	11 (10–12)	11 (11–12)
No school	669 (6.4)	1124 (13.5)	622 (6.5)	423 (3.7)	350 (3)
Primary education (1–7 years)	57 (0.5)	119 (1.4)	104 (1.1)	93 (0.8)	82 (0.7)
Secondary education (8–12 years)	2158 (20.7)	6488 (77.7)	8045 (84.5)	9997 (87.1)	10 223 (88.1)
Tertiary education (>12 Years)	156 (1.5)	495 (5.9)	421 (4.4)	574 (5)	672 (5.8)
Missing	7367 (70.8)	119 (1.4)	326 (3.4)	396 (3.4)	275 (2.4)
Current student	2660 (25.6)	1958 (23.5)	2475 (26)	2618 (22.8)	2064 (17.8)
Have/intend to return to school	2047 (19.7)	1461 (17.5)	1965 (20.6)	2208 (19.2)	1791 (15.4)
Unintended pregnancy	3959 (38)	4011 (48.1)	4453 (46.8)	4961 (43.2)	5177 (44.6)
Modern contraceptive use prior to pregnancy	2008 (19.3)	3142 (37.7)	3393 (35.6)	3414 (29.7)	2720 (23.4)
None	7419 (71.3)	4507 (54)	4196 (44.1)	5258 (45.8)	7220 (62.2)
Injectables	1282 (12.3)	2304 (27.6)	2684 (28.2)	2415 (21)	1631 (14.1)
Pills	712 (6.8)	796 (9.5)	607 (6.4)	640 (5.6)	579 (5)
Condoms	6 (0.1)	13 (0.2)	90 (0.9)	328 (2.9)	487 (4.2)
Other	41 (0.4)	69 (0.8)	43 (0.5)	38 (0.3)	47 (0.4)
Missing	947 (9.1)	656 (7.9)	1898 (19.9)	2804 (24.4)	1638 (14.1)
Using/intending to use modern postpartum contraception	3817 (36.7)	3286 (39.4)	3934 (41.3)	5987 (52.1)	7600 (65.5)
None	5339 (51.3)	4304 (51.6)	3564 (37.4)	2532 (22)	2180 (18.8)
Injectables	2863 (27.5)	2889 (34.6)	3445 (36.2)	5117 (44.6)	6520 (56.2)
Pills	618 (5.9)	324 (3.9)	338 (3.6)	401 (3.5)	627 (5.4)
Condoms	10 (0.1)	17 (0.2)	82 (0.9)	376 (3.3)	384 (3.3)
Emergency contraception	267 (2.6)	0 (0)	0 (0)	0 (0)	0 (0)
Sterilisation	47 (0.5)	46 (0.6)	42 (0.4)	33 (0.3)	26 (0.2)
Other	43 (0.4)	28 (0.3)	43 (0.5)	61 (0.5)	86 (0.7)
Missing	1220 (11.7)	737 (8.8)	2004 (21.1)	2963 (25.8)	1779 (15.3)
Delivery location					
Hospital	6596 (63.4)	5702 (68.3)	7226 (75.9)	9527 (83)	10 132 (87.3)
Clinic	632 (6.1)	486 (5.8)	605 (6.4)	493 (4.3)	377 (3.2)
Health centre	198 (1.9)	503 (6)	561 (5.9)	653 (5.7)	612 (5.3)
Home	2799 (26.9)	1492 (17.9)	910 (9.6)	548 (4.8)	298 (2.6)
Other	105 (1)	111 (1.3)	147 (1.5)	145 (1.3)	98 (0.8)
Missing	77 (0.7)	51 (0.6)	69 (0.7)	117 (1)	85 (0.7)
Birth attendant					
Doctor	813 (7.8)	838 (10)	923 (9.7)	1231 (10.7)	1176 (10.1)
Nurse	5406 (51.9)	5802 (69.5)	7331 (77)	9143 (79.6)	9920 (85.5)
Family member	1966 (18.9)	1018 (12.2)	682 (7.2)	397 (3.5)	190 (1.6)
Community member	330 (3.2)	212 (2.5)	86 (0.9)	78 (0.7)	43 (0.4)
Nobody	332 (3.2)	271 (3.2)	172 (1.8)	106 (0.9)	70 (0.6)
Other	0 (0)	0 (0)	0 (0)	0 (0)	3 (0)
Missing	1538 (14.8)	185 (2.2)	295 (3.1)	502 (4.4)	189 (1.6)
Nationality					
South African	6421 (61.7)	5431 (65.1)	6367 (66.9)	7892 (68.7)	8337 (71.9)
Mozambican	3962 (38.1)	2913 (34.9)	3139 (33)	3557 (31)	3189 (27.5)
Other	1 (0)	0 (0)	8 (0.1)	32 (0.3)	64 (0.6)
Missing	23 (0.2)	1 (0)	4 (0)	2 (0)	12 (0.1)
Residency status					
Permanent resident	8081 (77.6)	7436 (89.1)	7879 (82.8)	9088 (79.1)	9297 (80.1)
Temporary migrant	538 (5.2)	591 (7.1)	834 (8.8)	1461 (12.7)	1635 (14.1)
Other	43 (0.4)	256 (3.1)	574 (6)	660 (5.7)	522 (4.5)
Missing	1745 (16.8)	62 (0.7)	231 (2.4)	274 (2.4)	148 (1.3)

*Table 1 presents aggregate data across 5-year increments across the study period (the first column includes 6 instead of 5 years). This allowed for fewer instances of data aggregation into other categories when sparse data existed. Rows with fewer than five individuals in any group of years have been collapsed into the ‘other’ group in each category to protect participant privacy.

### Statistical analysis

We selected covariates for each model *a priori* such that all covariates preceded model outcomes and were informed from the literature and data availability (online supplemental etables 1–4).^1–4 21 22 25–27^ Antenatal care utilisation (count) was modelled with an ordinal logistic regression model (online supplemental etable 2). Skilled birth attendant presence at delivery (doctor, midwife or nurse vs other—binary) and delivery in a health facility (hospital, health centre or clinic vs other—binary) were modelled with logistic regression (online supplemental etable 3-4). Other skilled birth attendants included family members, community members or no one and other delivery locations included home deliveries and delivieries elsewhere in the community. Participants with missing outcome variables were excluded from each analysis (figure 2), whereas missing covariate data were imputed using multiple imputation with chained equations using 20 imputations for the primary analysis and included delivery year, age, antenatal visits, years of education, pregnancy number, student status, pregnancy intention, history of prepregnancy contraceptive use and type, nationality (South African, Mozambican, other) and residency status (permanent resident, temporary migrant and other status).^28^ All continuous covariates (age, antenatal visits, years of education and delivery year) were modelled as restricted cubic splines with four knots in the imputation (online supplemental etable 2–4).^28^

We calculated odds ratios for age at 10-year intervals from 15 to 45 years, for 0 vs 4 and 4 vs 8 antenatal visits, 0 vs 7 and 0 vs 12 years of education, and 1994 vs 2004 and 2004 vs 2018 for delivery year. Predictive models were validated and calibrated using 1000 bootstrapped samples to assess for model fit, discrimination, and, for the logistic regression models, calibration.^28^ All analyses were run in R Statistical Software and the code is available at https://github.com/dannysack/pregnancy_agincourt.^28–30^

### Sensitivity analyses

We conducted five *a priori* sensitivity analyses to assess the reliability of our model estimates among different subsets of our population. (1) Births that occurred 6 or fewer months before the observation date to account for potential recall bias that may arise the further a woman is interviewed from her delivery date (n=20 406). (2) Births that occurred 2 or fewer years before the observation date to exclude instances with a high likelihood of data entry error in line with our understanding of when the vast majority of births are captured (n=48 124). (3) Only full-term singleton births to account for recall impacted by unfavourable birth outcomes (n=50 138). (4) Only first recorded births to estimate effects in a potentially distinct population of first time mothers that are younger than the general Agincourt HDSS pregnant participants (n=36 008).^31^ (5) Only full-term singleton first recorded births to account for potential recall bias among first-recorded pregnancies (n=35 207). We also conducted one *post hoc* sensitivity analysis that included all women who delivered in 2001, 2003, 2005, 2007, 2009, 2011 and 2013–2018 (all the years with socioeconomic status data available) (n=25 711). We have no reason to believe that someone who delivered in 2001 is systematically different from someone who delivered in 2002, for example, and these estimates likely provide information on socioeconomic status’ influence on birth outcomes from 2001 to 2018. For computational efficiency, all sensitivity analyses used 10 imputations for missing data and excluded categorical prepregnancy contraception as an imputation covariate due to too few observations in some categories.^28^ In sensitivity analyses 4 and 5, we restricted our data to first recorded pregnancies and excluded pregnancy number from the imputation and models.

### Patient and public involvement

Participants and the public were not involved in the the design or analysis of this study.

## Results

Between 1993 and 2018 (table 1, figure 1 and online supplemental efigure 1), 51 355 pregnancies occurred, with fertility rates decreasing from 4.16 in 1993 to 1.90 in 2018, and the median number of antenatal visits increased steadily over time (table 1 and figure 1A). Skilled birth attendant presence and healthcare facility deliveries also increased steadily (table 1, online supplemental etable 2, figure 1B, C). Over the study period, unintended pregnancies remained around 50% (table 1 and online supplemental efigure 1), whereas the percentage of those intending to use/or currently using postpartum contraceptives increased over time, predominantly via injectable contraceptives (table 1 and figure 1D). From 1993 to 1998, almost 40% of deliveries—compared with 27% of deliveries from 2014 to 2018—were among mothers of Mozambican origin (table 1). Deliveries among temporary migrants increased from 5.2% of deliveries (1993–1998) to 14.1% of deliveries (2014–2018, table 1) as compared with Agincourt permanent residents, which remained relatively stable around 80% across the study period, reflecting the increased prevalence of temporary migration among early adult women over time.^32^ Other pregnancy characteristics, such as age at delivery (median ranged from 24.6 to 25.8 across all time periods) and years of education at delivery (median ranged from 10 to 11 years across all time periods) remained fairly constant over the study period (table 1, online supplemental efigure 1). Other descriptive covariates of interest are displayed in table 1, figure 1, online supplemental efigure 1.

**Figure 3:**
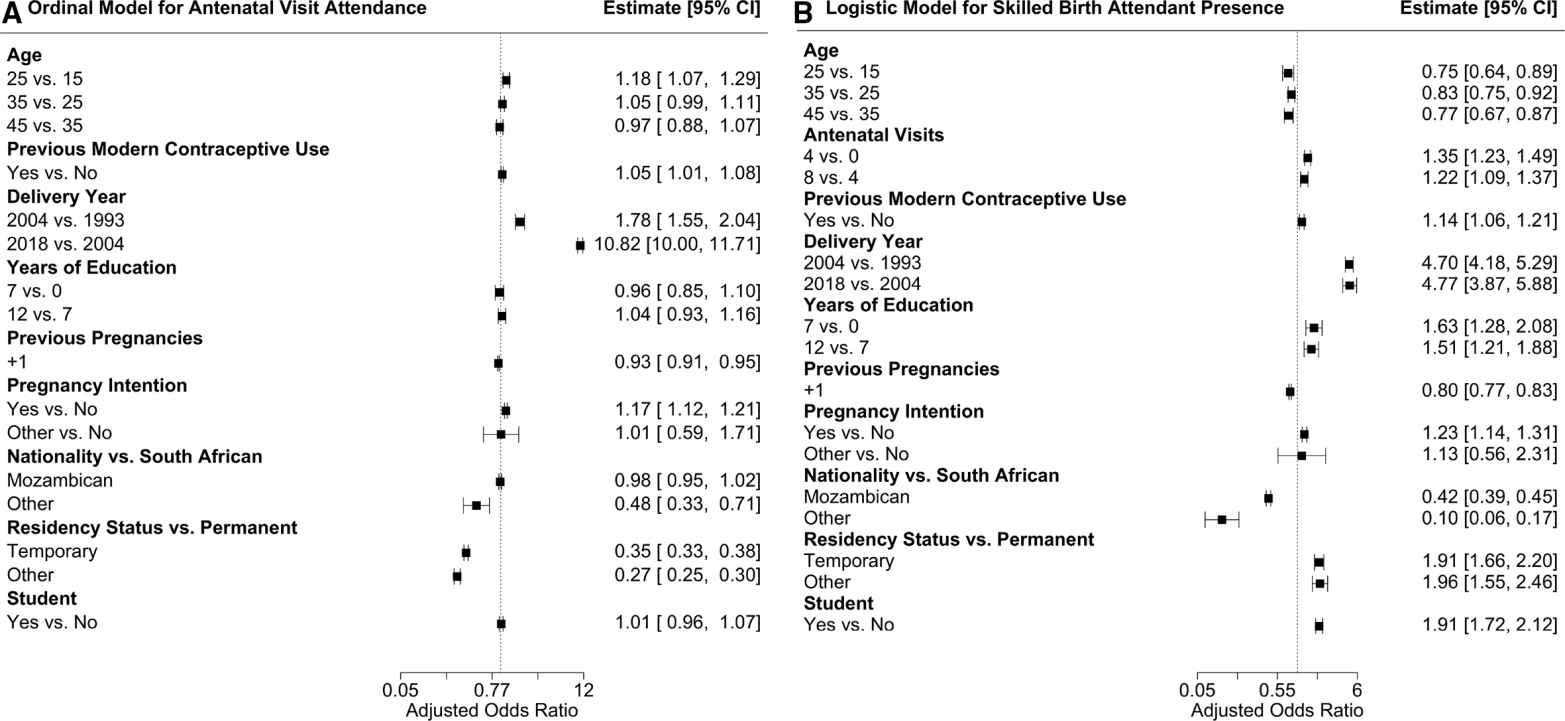
Predictive model forest plots. It shows forest plots for each covariate in (A) the ordinal model for antenatal visit attendance and (B) the logistic model for skilled birth attendant presence. Both plots present the adjusted OR (estimate) and the 95% CI for each covariate. Information the logistic model for health facility delivery is presented in online supplemental efigure 3.

**Figure 4:**
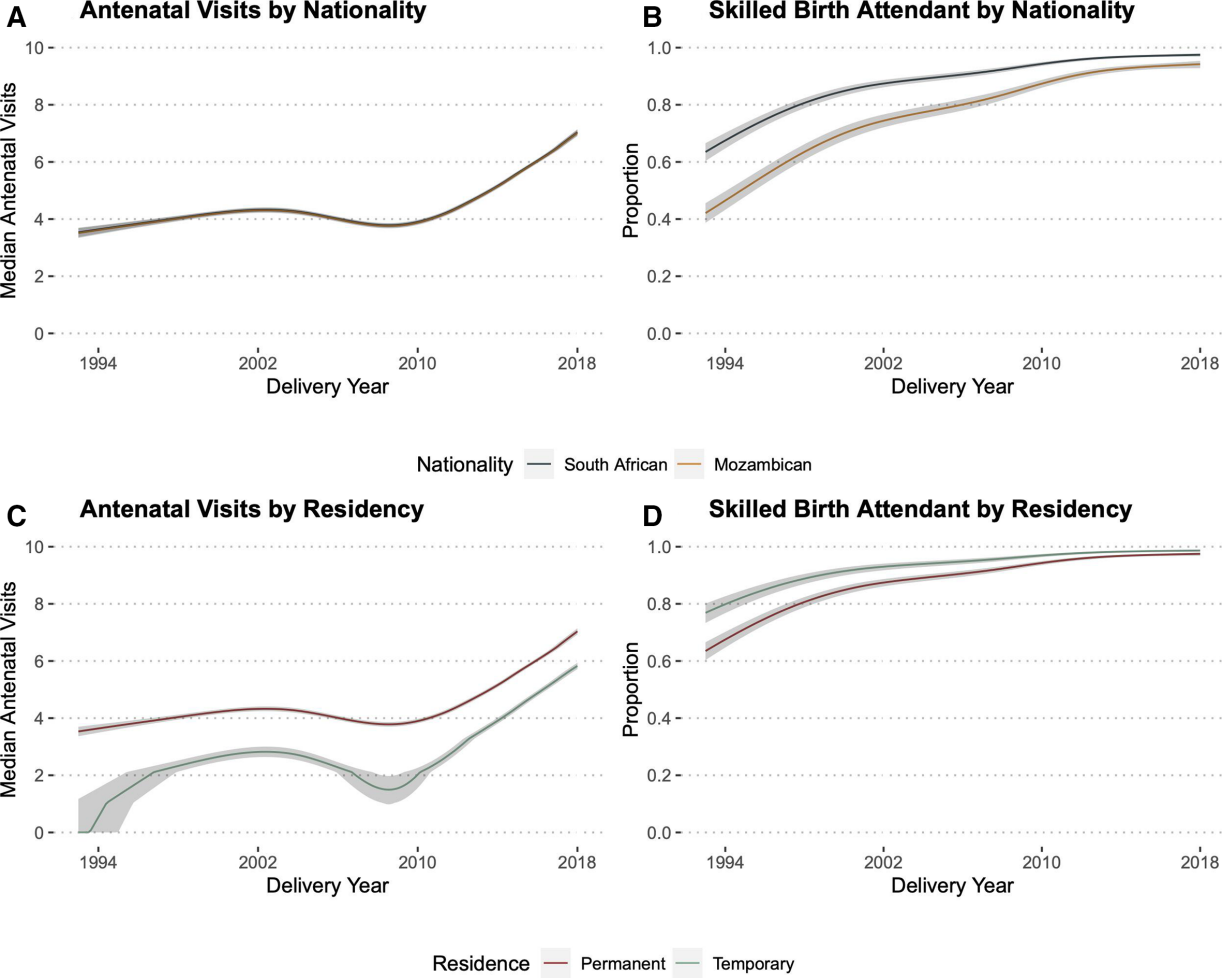
Antenatal visits and skilled birth attendant by delivery year, nationality and residency status. It show partial effects plots—which allow for visualisation of an outcome of interest adjusted for covariates of interest—with predicted median antenatal visits (A, C) and proportion of births with a skilled birth attendant (B, D) over time. Each plot was adjusted for age at delivery, number of antenatal visits, previous modern contraceptive use, years of education, number of previous pregnancies, pregnancy intention and student status. The proportion of deliveries at a health facility over time are presented in online supplemental efigure 3. Note that in the absence of an interaction term, the lines are forced to be parallel. Although mothers of Mozambique descent had a similar median predicted number of antenatal visits compared with South Africans (A), they were less likely to have a skilled birth attendant at delivery (B) compared with South Africans as late as 2018. Despite fewer median antenatal visits among participants who identified as temporary migrants as compared with permanent residents (C), temporary migrants were more likely to have a skilled birth attendant present at delivery (D). We excluded ‘other’ from this figure because of the very small group of deliveries among individuals who identified as ‘other’ nationality or residence status, limiting the interpretation of the predicted proportions from each model (table 1).

In modelling median antenatal visits, delivery year, residency status and pregnancy intention were clinically meaningful predictors of an additional antenatal visit among study participants (figures 3A, 4A, C). Delivery in 2018, for example, was associated with a 10.82 times (95% CI 10.0 to 11.71) increased likelihood of one or more additional antenatal visits as compared with delivery in 2004. Temporary migrant participants had a decreased likelihood of one or more additional antenatal visits compared with permanent Agincourt residents (adjusted OR (aOR) 0.35; 95% CI 0.33 to 0.38) (figure 4C). Intended pregnancies were associated with an increased likelihood of one or more additional antenatal visits compared with unintended pregnancies (aOR 1.17; 95% CI 1.12 to 1.21). The proportional odds assumption also appears to hold for all included covariates until rare high numbers of antenatal visits (>12) (online supplemental efigure 2).

Delivery year, nationality and residency status were important predictors of women having a skilled birth attendant (figures 3B, 4B, C). Delivery in 2018 was associated with a 4.77 times (95% CI 3.87 to 5.88) increased likelihood of skilled birth attendant presence as compared with delivery in 2004 and delivery in 2004 was associated with a 4.7 times (95% CI 4.18 to 5.29) increased likelihood as compared with 1994. Women of Mozambican origin had a lower likelihood of skilled birth attendant presence as compared with South Africans (aOR 0.42, 95% CI 0.39 to 0.45) and women identified as temporary migrants had a higher likelihood of skilled birth attendance than permanent residents (aOR 1.91, 95% CI 1.66 to 2.20), although these differences decreased over time (figure 4B, D). Delivery year, nationality and residency status were also key predictors of health facility delivery (online supplemental etable 6 and efigure 3). The skilled birth attendant and health facility delivery models had good discrimination and calibration characteristics (online supplemental etable 7 and efigure 4).

Since delivery year was the most important predictor in all three models (described above), we examine predicted medians and proportions, respectively, via partial effects plots. As time progressed, the median number of antenatal visits and the proportion of deliveries staffed with a skilled birth attendant and at a health facility increased differentially by nationality and residency status (figure 4, online supplemental efigure 3). Finally, the direction and magnitude of the predictors did not change notably across all five sensitivity analyses (online supplemental etable 8–10 and efigure 5). In our sensitivity analyses that included absolute household socioeconomic status, the direction and magnitude of the other variables did not change, although it did marginally temper the negative association between being from Mozambique and delivering with a skilled birth attendant (aOR 0.7, 95% CI 0.62 to 0.79) (online supplemental etable 8–10 and efigure 5). A one-unit increase in absolute household socioeconomic status was associated with an increased likelihood of attending an additional antenatal visit (aOR 1.1, 95% CI 1.04 to 1.17) and delivering with a skilled birth attendant (aOR 1.92, 95% CI 1.67 to 2.22).

## Discussion

These data show how the characteristics of pregnant woman and their pregnancy-related healthcare utilisation have evolved in a rural South African population over a period marked by substantial sociopolitical and epidemiological transitions. The descriptive trends align with South African Demographic and Health Survey (DHS) data as far back as 1998.^22 23 33^ DHS data on antenatal visits follow a similar U-shaped trend to our data, while the likelihood of skilled birth attendant presence and delivery at a health facility increase linearly throughout the study period.^22^ These data suggest that South Africa’s efforts to improve maternal health and maternity care have successfully increased pregnancy-related healthcare utilisation in rural Mpumalanga—at least with respect to antenatal visits, skilled birth attendant presence and health facility delivery.^5–7 14^ This study cannot comment on the specific components of the strategic plan, or concurrent policy changes, which may have driven these changes; however, it does highlight fruitful avenues for future research, specifically examining pregnancy-related outcomes among non-South Africans and temporary migrants in the region.

The overall improvements appear to have continued in spite of concerns that in the aftermath of apartheid, economic growth policies exacerbated existing structural healthcare inequities.^8 34^ Although the decrease in antenatal visits in the early 2000s could reflect a health system overwhelmed with and struggling to respond to HIV/AIDS and its associated stigma,^7 15 16^ more recent trends are promising. These advances may reflect better access to health facilities secondary to a combination of improved infrastructure, health system improvements, patient socioeconomic status or the 1994 abolition of user fees for pregnant and lactating women.^35^ Recent iterations of South Africa’s maternity care guidelines have advocated for prenatal care’s integration into primary health clinics, succinct checklists for antenatal care visits, and improved services and staffing at primary health clinics.^6 14 36^

There seems to be an increase in the intention to use/current postpartum contraceptive use across the study period, particularly due to increased injectable contraceptive uptake, which also aligns with decreased fertility rates in this population.^37–39^ While this follows WHO best practices and the South African strategic plan,^5 40^ it is essential to consider these findings in the context of South Africa’s contraceptive history. During apartheid, the government’s contraceptive priorities were motivated by the racist desire to limit reproduction in Black South Africans.^7 41 42^ There was, therefore, robust infrastructure to provide long-acting injectable contraceptives in homelands, despite poor access to other contraceptive methods or primary and maternal health services.^7 41 42^ The end of apartheid, in addition to a new focus on comprehensive primary and maternal care services, brought a new population policy focused on shared decision making and women’s empowerment.^7 43^ It is worth considering how provider bias, which can stem from practice norms and historical structures, may impact how patients are counselled on their contraceptive options.^44^

There also appear to be differential levels of skilled birth attendant presence and health facility delivery among participants born in South Africa versus those of Mozambican origin, and among permanent HDSS residents versus temporary migrants. Although mothers of Mozambican descent were equally likely to attend antenatal visits, they were less likely to deliver with a skilled birth attendant or in a health facility. While a mother of Mozambican descents’ fertility trends have converged with local patterns,^37–39^ their pregnancy-related healthcare utilisation has not. This aligns with previous studies in the Agincourt area, showing that people of Mozambican origin experience worse health outcomes due to their lower wealth and perceived lack of legal status.^45 46^ Additionally, evidence from South Africa and other low-to-middle-income countries suggests limited access to health facilities among immigrant populations.^47 48^ Our results suggest that, while differences are shrinking, there may be insufficient support for non-South African mothers living in the Agincourt HDSS catchment area. The specificity of decreased utilisation in hospital-based services (as opposed to antenatal services) could stem from documented xenophobia in the South African health system, which may result in the reduced utilisation of health services by immigrant mothers^49 50^ or, in the Agincourt context, socioeconomic differences, particularly limited asset accumulation or differential access to social services.^46 51^

While temporary migrants reported fewer antenatal visits than permanent residents, they were more likely to report skilled birth attendant presence and delivery at a health facility than permanent Agincourt residents. We would expect a disruption in antenatal services due to migration,^45^ particularly given the circular, temporary migration prevalent in the Agincourt population. A study of health utilisation among temporary migrants from this sub-district found migrants with chronic conditions were less likely than permanent Agincourt residents to use health services.^32^ However, migrants may be more inclined to use private health services and report current employment, suggesting that they may have more resources to commit to a health facility delivery.^32^ Further, it is possible that being detached from their households, migrants may have less family and community support, thus motivating a health facility delivery. Finally rural South Africans face greater cost and access barriers when seeking obstetric care than urban South Africans,^52^ and temporary migrants in the Agincourt HDSS tend to work in urban areas.^32^

### Limitations

These data and results are subject to several limitations. They are prone to respondent biases, either social desirability or recall bias related to events that occurred during a woman’s pregnancy. However, these data are collected and updated annually, which may limit recall bias. While measurement error in our covariates and outcomes were likely random and therefore non-differential, since some covariates included multiple categories it is unclear whether biases cancelled out all together or were towards or away from the null. Our five sensitivity analyses suggest that our conclusions are robust to several selection criteria. Furthermore, while we could only use Agincourt HDSS household asset-based socioeconomic status data in a *post hoc* sensitivity analysis due to how we extracted the data, it did not meaningfully change our model coefficients. Despite the large sample size, small strata exist within some covariates and although these data may be reflective of rural regions of South Africa, it is difficult to apply the results to more urban areas or other populations. While we can assume temporality between some covariates (eg, antenatal visits coming before delivery), we could not infer causality and were limited to predictive models that strictly provide information about the association between covariates and outcomes of interest. Future studies should use the Agincourt HDSS’s longitudinal design and robust follow-up to ask causal questions about the relationship between pregnancy-related healthcare utilisation and nationality and/or residency status.

## Conclusion

These population-level data can inform decisions about maternal health policy in rural northeastern South Africa and may be applicable to demographically similar regions of South Africa. Our findings provide important insights into how pregnancy-related care, specifically healthcare utilisation, has changed over the course of the last 30 years in rural South Africa, which aligns with multifaceted changes in South African health policy from the end of apartheid through the HIV epidemic. Future research, which should include data that documents changes in health service availability and utilisation, should focus on health services utilisation during pregnancy among foreign national and temporary migrant mothers to further examine why they seem less likely to use pregnancy-related healthcare.

## Data Availability

Data are available on reasonable request, after approval of any requisite Agincourt Health and Demographic Surveillance Systems processes. All data cleaning and analysis code are available at https://github.com/dannysack/pregnancy_agincourt.
