# Supplementary material for: Pregnancy-related healthcare utilisation in Agincourt, South Africa, 1993–2018: a longitudinal surveillance study of rural mothers
Source: BMJ Glob Health. 2021 Oct 7;6(10):e006915. doi: 10.1136/bmjgh-2021-006915 (PMC8499259; doi:10.1136/bmjgh-2021-006915)
Supplement: Supplementary data [file bmjgh-2021-006915supp001.pdf]

Supplementary Tables and Figures

eTable 1: Variables of Interest			
**AHDSS Table**	**Field Description/*Survey Question***	**Field Name**	**Data Type**
Observation	Indicates the date on which this observation took place	ObservationDate	Numeric
Observation	Observation code	Observation	Numeric
Pregnancies	Unique ID associated with this pregnancy	Pregnancy	Numeric
Pregnancies	Observation code	Observation	Numeric
Pregnancies	Indicates whether mother attended antenatal clinic while pregnant	AntenatalClinic	Text (Binary)
Pregnancies	*How many times did you visit an antenatal clinic while you were pregnant?*	AntenatalVisits	Numeric
Pregnancies	*What was you last grade/standard completed?*	Education	Text (Categorical)
Pregnancies	*Were you a student at the time you became pregnant?*	Scholar	Text (Binary)
Pregnancies	*Are you going back to school?*	BackToSchool	Text (Binary)
Pregnancies	*Was this pregnancy planned?*	PregnancyPlanned	Text (Binary)
Pregnancies	*Did you use any type of contraceptive at any time prior to this pregnancy?*	ContraceBe	Text (Categorical)
Pregnancies	*Which kind of contraception are you using or intending to use after pregnancy?*	ContraceAf	Text (Categorical)
Pregnancies	Date of delivery	DeliveryDate	Numeric
Pregnancies	*Delivery in Agincourt area?*	DeliveryLocal	Text (Binary)
Pregnancies	*Where did you deliver?*	DeliveryPlace	Text (Categorical)
Pregnancies	*Which hospital?*	Hospital	Text (Categorical)
Pregnancies	*Who attended the delivery?*	Attendant	Text (Categorical)
Pregnancies	*Was there a complication at delivery?*	Complication	Text (Categorical)
Pregnancies	Indicates the outcome of the pregnancy	Outcome	Text (Categorical)
Pregnancies	Number of babies still born for this pregnancy	StillBorn	Numeric
Pregnancies	Number of babies live born for this pregnancy	LiveBorn	Numeric
Individuals	Individual's birth date	DoB	Numeric
Individuals	Indicates whether the individual is a refugee	Refugee	Text (Categorical)
Births	Number to link to the pregnancy field in the pregnancies table	Pregnancy	Numeric
ResidentStatus	Residency status of the individual	ResStatus	Text (Categorical)
SESIndex	Composite socioeconomic status indicator	SES_absolute	Numeric

Age at delivery was calculated from the mother's date of birth (DoB) and the date of delivery (DeliveryDate). Calculated ages less than 11 and greater than 55 were assumed to be secondary to data entry errors and marked as missing. We imputed zero antenatal visits (AntenatalVisits) to the 610 cases where AntenatalVisits was missing, but AntenatalClinic was "No" for all analyses. AntenatalVisits values

greater than 15 were assumed to be data error errors and coded as missing. We coded Education as a numerical value, which allowed us to standardize years of reported South African and Mozambican school, as well as adult educational programs such as the adult basic education and training and national qualification framework in South Africa to their equivalent years of education. The proportion of women using contraception before and after pregnancy was presented by method and dichotomized to use modern contraceptive methods or not (modern contraceptive include: condom use, emergency contraceptive use, injectable use, loop use, more than one contraceptive use, long acting reversible contraceptive use, pill use, and sterilization) for Table 1 and some imputations as described in the methods.[1] Pregnancy intention was coded as intended, unintended, or other, and student status was dichotomous (yes vs. no), all were unchanged from the AHDSS dataset. Nationality was coded as South African, Mozambican (combined pre- and post-1992 arrival), and other (any other country) and migrant status was coded as resident (lived more than six months in the area over the previous year), temporary migrant (lived fewer than six months in the area), and other migrant (in the study area for education, to care for a family member, or another reason). Time between delivery and observation date was calculated as days between the delivery date and the observation date for sensitivity analyses. Absolute household socioeconomic status, which was only collected in every other year from 2001 to 2013 and yearly from 2014 onwards, is a composite score that accounts for dwelling, sanitation, power, modernity, and livestock assets in each included household. Kabudula et al. (2017) describes measuring socioeconomic status in this cohort in greater detail.[2]

eTable 2: Ordinal Logistic Model for Antenatal Visit Attendance		
**Predictor**	**Variable Form**	**Degrees of Freedom**
Age	Restricted Cubic Spline, 5 knots	4
Previous Modern Contraceptive Use	Dichotomous	1
Delivery Year	Restricted Cubic Spline, 5 knots	4
Years of Education	Restricted Cubic Spline, 5 knots	4
Previous Pregnancies	Linear	1
Unintended Pregnancy	Categorical	2
Yes		
Other		
Nationality vs. South African	Categorical	2
Mozambican		
Other		
Residency Status vs. Permanent	Categorical	2
Temporary		
Other		
Current Student	Dichotomous	1

eTable 3: Logistic Model for Skilled Birth Attendant Presence		
**Predictor**	**Variable From**	**Degrees of Freedom**
Age	Restricted Cubic Spline, 5 knots	4
Antenatal Visits	Restricted Cubic Spline, 5 knots	4
Previous Modern Contraceptive Use	Dichotomous	1
Delivery Year	Restricted Cubic Spline, 5 knots	4
Years of Education	Restricted Cubic Spline, 5 knots	4
Previous Pregnancies	Linear	2
Unintended Pregnancy	Categorical	2
Yes		
Other		
Migration Status vs. South African	Categorical	2
Mozambican		
Other		
Residency Status vs. Permanent	Categorical	2
Temporary		
Other		
Current Student	Dichotomous	1

eTable 4: Logistic Model for Health Facility Delivery

Predictor		Variable From	Degrees of Freedom
Age		Restricted Cubic Spline, 5 knots	4
Antenatal Visits		Restricted Cubic Spline, 5 knots	4
Previous Modern Contraceptive Use		Dichotomous	1
Delivery Year		Restricted Cubic Spline, 5 knots	4
Years of Education		Restricted Cubic Spline, 5 knots	4
Previous Pregnancies		Linear	2
Unintended Pregnancy		Categorical	2
	Yes		
	Other		
Migration Status vs. South African		Categorical	3
	Mozambican		
	Other		
Residency Status vs. Permanent		Categorical	2
	Temporary		
	Other		
Current Student		Dichotomous	1

eTable 5. Missing data by analysis

n (%) or median (interquartile range)		Antenatal Visits		Skilled Birth Attendant		Health Facility Delivery	
		Included	Excluded	Included	Excluded	Included	Excluded
Pregnancies		**42878**	**8477**	**48646**	**2709**	**50956**	**399**
	First Time Pregnancies	28901 (67.4)	7107 (83.8)	33723 (69.3)	2285 (84.3)	35749 (70.2)	259 (64.9)
Age		25.0 (20.3-31.0)	25.4 (20.3-31.5)	25.1 (20.3-31.1)	25.3 (20.3-31.3)	25.1 (20.3-31.1)	26.7 (22.4-32.0)
	Missing	17.0 (0.0)	39.0 (0.5)	49.0 (0.1)	7.00 (0.3)	56.0 (0.1)	0 (0)
Median Antenatal Visits		4.00 (3.00-6.00)	—	5.00 (3.00-6.00)	0 (0-0)	4.00 (3.00-6.00)	0 (0-5.00)
	Missing	0 (0)	8477 (100)	6919 (14.2)	1558 (57.5)	8376 (16.4)	101 (25.3)
Median Year of Education		11.0 (10.0-12.0)	12.0 (11.0-12.0)	11.0 (10.0-12.0)	11.0 (10.0-12.0)	11.0 (10.0-12.0)	11.0 (10.0-12.0)
	Missing	1198 (2.8)	7285 (85.9)	6814 (14.0)	1669 (61.6)	8378 (16.4)	105 (26.3)
Current Student							
	Have/Intend to Return to School	9751 (22.7)	2024 (23.9)	11120 (22.9)	655 (24.2)	11723 (23.0)	52.0 (13.0)
Unintended Pregnancy		19632 (45.8)	2929 (34.6)	21824 (44.9)	737 (27.2)	22468 (44.1)	93.0 (23.3)
Modern Contraceptive Use Prior to Pregnancy		13554 (31.6)	1123 (13.2)	14404 (29.6)	273 (10.1)	14624 (28.7)	32 (13.3)
	None	22834 (53.3)	5766 (68.0)	27317 (56.2)	1283 (47.4)	28493 (55.9)	107 (26.8)
	Injectables	9659 (22.5)	657 (7.8)	10146 (20.9)	170 (6.3)	10284 (20.2)	32 (8.0)
	Pills	2894 (6.7)	440 (5.2)	3241 (6.7)	93.0 (3.4)	3318 (6.5)	16 (4.0)
	Condoms	905 (2.1)	19.0 (0.2)	918 (1.9)	6.00 (0.2)	921 (1.8)	—
	Other	208 (0.5)	30 (0.4)	227 (0.5)	11 (0.4)	235 (0.5)	6 (1.5)
	Missing	6378 (14.9)	1565 (18.5)	6797 (14.0)	1146 (42.3)	7705 (15.1)	238 (59.6)
Using/Intending to use Modern Postpartum Contraception		22242 (51.9)	2382 (28.1)	24167 (49.7)	457 (16.9)	24548 (48.2)	76 (19.0)
	None	13715 (32.0)	4204 (49.6)	16957 (34.9)	962 (35.5)	17845 (35.0)	74.0 (18.5)
	Injectables	19205 (44.8)	1629 (19.2)	20498 (42.1)	336 (12.4)	20771 (40.8)	63.0 (15.8)
	Pills	1875 (4.4)	433 (5.1)	2205 (4.5)	103 (3.8)	2299 (4.5)	9.00 (2.3)
	Condoms	852 (2.0)	17 (0.2)	866 (1.8)	—	866 (1.7)	—
	Emergency Contraception	—	263 (3.1)	266 (0.5)	—	267 (0.5)	—
	Sterilization	165 (0.4)	29 (0.3)	185 (0.4)	9 (0.3)	193 (0.4)	—
	Other	236 (0.6)	29 (0.3)	248 (0.5)	17 (0.6)	260 (0.5)	5 (1.3)
	Missing	6830 (15.9)	1873 (22.1)	7421 (15.3)	1282 (47.3)	8455 (16.6)	248 (62.2)
Delivery Location							
	Hospital	33548 (78.2)	5635 (66.5)	37157 (76.4)	2026 (74.8)	39183 (76.9)	0 (0)
	Clinic	2013 (4.7)	580 (6.8)	2424 (5.0)	169 (6.2)	2593 (5.1)	0 (0)
	Health Center	2484 (5.8)	43.0 (0.5)	2495 (5.1)	32.0 (1.2)	2527 (5.0)	0 (0)

	Home	4018 (9.4)	2029 (23.9)	5854 (12.0)	193 (7.1)	6047 (11.9)	0 (0)
	Other	517 (1.2)	89 (1.0)	528 (1.1)	78.0 (2.9)	606 (1.2)	0 (0)
	Missing	298 (0.7)	101 (1.2)	188 (0.4)	211 (7.8)	0 (0)	399 (100)
Birth Attendant							
	Doctor	4268 (10.0)	713 (8.4)	4981 (10.2)	0 (0)	4960 (9.7)	21.0 (5.3)
	Nurse	33280 (77.6)	4325 (51.0)	37605 (77.3)	0 (0)	37465 (73.5)	140 (35.1)
	Family Member	2819 (6.6)	1434 (16.9)	4253 (8.7)	0 (0)	4238 (8.3)	15.0 (3.8)
	Community Member	528 (1.2)	221 (2.6)	749 (1.5)	0 (0)	745 (1.5)	4.00 (1.0)
	Nobody	741 (1.7)	210 (2.5)	951 (2.0)	0 (0)	949 (1.9)	—
	Other	91 (0.2)	16 (0.2)	107 (0.2)	0 (0)	101 (0.2)	8 (2.0)
	Missing	1151 (2.7)	1558 (18.4)	0 (0)	2709 (100)	2498 (4.9)	211 (52.9)
Nationality							
	South African	29125 (67.9)	5323 (62.8)	32573 (67.0)	1875 (69.2)	34161 (67.0)	287 (71.9)
	Mozambican	13643 (31.8)	3117 (36.8)	15938 (32.8)	822 (30.3)	16651 (32.7)	109 (27.3)
	Other	96 (0.2)	9 (0.1)	99 (0.2)	6 (0.2)	102 (0.2)	3 (0.8)
	Missing	14 (0.0)	28 (0.3)	36 (0.1)	6 (0.2)	42 (0.1)	0 (0)
Residency Status							
	Permanent Resident	35741 (83.4)	6040 (71.3)	40972 (84.2)	809 (29.9)	41534 (81.5)	247 (61.9)
	Temporary Migrant	4433 (10.3)	626 (7.4)	4675 (9.6)	384 (14.2)	4966 (9.7)	93 (23.3)
	Other	1908 (4.4)	147 (1.7)	1857 (3.8)	198 (7.3)	2011 (3.9)	44 (11.0)
	Missing	796 (1.9)	1664 (19.6)	1142 (2.3)	1318 (48.7)	2445 (4.8)	15 (3.8)

Rows with fewer than five individuals in any group of years have been collapsed into the "Other" group in each category to protect participant privacy.

**eTable 6. Predictive Models of Pregnancy-Related Health Outcomes**				
		**Antenatal Visits**	**Skilled Birth Attendant**	**Health Facility Delivery**
		aOR (95% CI)	aOR (95% CI)	aOR (95% CI)
Age				
	25 vs. 15	1.18 (1.07, 1.29)	0.75 (0.64, 0.89)	0.74 (0.63, 0.86)
	35 vs. 25	1.05 (0.99, 1.11)	0.83 (0.75, 0.92)	0.81 (0.74, 0.89)
	45 vs. 35	0.97 (0.88, 1.07)	0.77 (0.67, 0.87)	0.81 (0.72, 0.92)
Antenatal Visits				
	4 vs. 0	—	1.22 (1.09, 1.37)	1.13 (1.01, 1.27)
	8 vs. 4	—	1.35 (1.23, 1.49)	1.3 (1.19, 1.43)
Previous Modern Contraceptive Use				
	Yes vs. No	1.05 (1.01, 1.08)	1.14 (1.06, 1.21)	1.17 (1.1, 1.25)
Delivery Year				
	2004 vs. 1993	1.78 (1.55, 2.04)	4.7 (4.18, 5.29)	2.77 (2.49, 3.07)
	2018 vs. 2004	10.82 (10.0, 11.71)	4.77 (3.87, 5.88)	4.05 (3.39, 4.84)
Years of Education				
	7 vs. 0	0.96 (0.85, 1.1)	1.63 (1.28, 2.08)	1.67 (1.34, 2.08)
	12 vs. 7	1.04 (0.93, 1.16)	1.51 (1.21, 1.88)	1.32 (1.07, 1.62)
Previous Pregnancies				
	+1	0.93 (0.91, 0.95)	0.8 (0.77, 0.83)	0.82 (0.79, 0.85)
Pregnancy Intention				
	Yes vs. No	1.17 (1.12, 1.21)	1.23 (1.14, 1.31)	1.15 (1.08, 1.23)
	Other vs. No	1.01 (0.59, 1.71)	1.13 (0.56, 2.31)	1.47 (0.86, 2.51)
Nationality vs. South African				
	Mozambican	0.98 (0.95, 1.02)	0.42 (0.39, 0.45)	0.43 (0.41, 0.46)
	Other	0.48 (0.33, 0.71)	0.1 (0.06, 0.17)	0.14 (0.08, 0.22)
Residency Status vs. Permanent				
	Temporary	0.35 (0.33, 0.38)	1.91 (1.66, 2.2)	1.40 (1.24, 1.58)
	Other	0.27 (0.25, 0.3)	1.96 (1.55, 2.46)	1.47 (1.21, 1.77)
Student				
	Yes vs. No	1.01 (0.96, 1.07)	1.91 (1.72, 2.12)	1.81 (1.64, 1.99)

Antenatal Visits was an ordinal logistic model, whereas Skilled Birth Attendant and Health Facility Delivery were predicted with a binary logistic model.

**eTable 7: Model Characteristics**			
	**Antenatal Visits**	**Skilled Birth Attendant**	**Health Facility Delivery**
Sample size	42,878	48,646	50,956
Events	—	42,586	44,303
Degrees of freedom	21	25	25
Events per variable	—	1,703	1,772
Likelihood ratio X^2	8,478.34	7,629.47	6,830.09
Probability (> X^2)	<0.0001	<0.0001	<0.0001
C, statistic	—	0.816	0.789
rho	0.385	—	—
Optimism-corrected rho*	0.384	—	—
Slope	1	1	1
Optimism-corrected slope*	0.998	0.997	0.995
Brier score	—	0.088	0.095
Optimism-corrected Brier score*	—	0.088	0.095
Mean absolute error	—	0.003	0.007
Mean squared error	—	0.00002	0.00006

*1000 bootstrapped repetitions

eTable 8: Sensitivity Analyses for Antenatal Visits Model

		Primary analysis	Births < 6 months before observation date	Births < 2 years before observation date	Full singleton births	First recorded births	Full singleton first recorded births	Absolute household socioeconomic status available
n		42,878	17,779	40,277	41,747	28,901	28,176	25,711
Age								
	25 vs. 15	1.18 (1.07, 1.29)	1.19 (1.02, 1.37)	1.19 (1.08, 1.31)	1.17 (1.07, 1.29)	1.24 (1.12, 1.37)	1.22 (1.1, 1.36)	1.2 (1.05, 1.36)
	35 vs. 25	1.05 (0.99, 1.11)	1.1 (1.01, 1.21)	1.04 (0.98, 1.11)	1.06 (1, 1.13)	1.03 (0.97, 1.1)	1.05 (0.98, 1.12)	1.03 (0.96, 1.11)
	45 vs. 35	0.97 (0.88, 1.07)	1 (0.86, 1.16)	0.96 (0.87, 1.07)	0.98 (0.89, 1.08)	0.96 (0.85, 1.08)	0.96 (0.85, 1.08)	0.96 (0.84, 1.1)
Previous Modern Contraceptive Use								
	Yes vs. No	1.05 (1.01, 1.08)	1.01 (0.95, 1.07)	1.02 (0.98, 1.06)	1.02 (0.98, 1.06)	1.02 (0.97, 1.07)	1.01 (0.96, 1.06)	0.99 (0.93, 1.04)
Delivery Year								
	2004 vs. 1993	1.78 (1.55, 2.04)	4.03 (3.07, 5.29)	2.22 (1.88, 2.62)	1.58 (1.37, 1.82)	1.85 (1.57, 2.17)	1.64 (1.39, 1.94)	—
	2018 vs. 2004	10.82 (10, 11.71)	8.69 (7.69, 9.82)	10.38 (9.57, 11.25)	10.85 (10.01, 11.75)	10.12 (9.18, 11.16)	10.17 (9.22, 11.23)	10 (9.05, 11.04)
Years of Education								
	7 vs. 0	0.96 (0.85, 1.1)	0.85 (0.7, 1.04)	0.97 (0.84, 1.11)	0.96 (0.84, 1.09)	1.07 (0.91, 1.26)	1.03 (0.87, 1.22)	0.74 (0.62, 0.88)
	12 vs. 7	1.04 (0.93, 1.16)	1.13 (0.95, 1.34)	1.04 (0.92, 1.17)	1.05 (0.94, 1.18)	1.06 (0.92, 1.22)	1.09 (0.95, 1.25)	1.11 (0.95, 1.28)
Previous Pregnancies								
	+1	0.93 (0.91, 0.95)	0.92 (0.89, 0.95)	0.94 (0.92, 0.96)	0.93 (0.91, 0.95)	—	—	0.92 (0.9, 0.95)
Pregnancy Intention								
	Yes vs. No	1.17 (1.12, 1.21)	1.19 (1.11, 1.26)	1.16 (1.12, 1.21)	1.17 (1.12, 1.22)	1.12 (1.07, 1.18)	1.14 (1.08, 1.2)	1.15 (1.09, 1.22)
	Other vs. No	1.01 (0.59, 1.71)	0.79 (0.27, 2.35)	0.92 (0.56, 1.51)	0.98 (0.58, 1.65)	0.83 (0.43, 1.58)	0.96 (0.5, 1.84)	1.01 (0.55, 1.89)
Nationality vs. South African								
	Mozambican	0.98 (0.95, 1.02)	0.96 (0.91, 1.02)	0.98 (0.94, 1.02)	0.97 (0.93, 1.01)	1 (0.95, 1.05)	0.98 (0.94, 1.03)	1.02 (0.97, 1.07)
	Other	0.48 (0.33, 0.71)	0.56 (0.32, 1.01)	0.44 (0.3, 0.66)	0.47 (0.31, 0.69)	0.51 (0.33, 0.78)	0.49 (0.32, 0.76)	0.45 (0.29, 0.69)
Residency Status vs. Permanent								
	Temporary	0.35 (0.33, 0.38)	0.43 (0.38, 0.47)	0.36 (0.34, 0.38)	0.35 (0.33, 0.37)	0.37 (0.35, 0.4)	0.37 (0.34, 0.4)	0.44 (0.41, 0.48)
	Other	0.27 (0.25, 0.3)	0.25 (0.22, 0.29)	0.27 (0.24, 0.29)	0.27 (0.25, 0.3)	0.31 (0.27, 0.34)	0.31 (0.28, 0.34)	0.34 (0.3, 0.38)
Student								
	Yes vs. No	1.01 (0.96, 1.07)	0.99 (0.91, 1.09)	1.01 (0.95, 1.07)	1.01 (0.95, 1.07)	1.01 (0.95, 1.07)	1 (0.94, 1.07)	0.96 (0.89, 1.04)
Absolute household socioeconomic status								
	+1 increase in socioeconomic status index	—	—	—	—	—	—	1.1 (1.04, 1.17)

eTable 9: Sensitivity Analyses for Skilled Birth Attendant Model		Primary analysis	Births < 6 months before observation date	Births < 2 years before observation date	Full singleton births	First recorded births	Full singleton first recorded births	Absolute household socioeconomic status available
n		48,646	19,239	45,592	47,526	33,723	33,000	25,711
Age								
	25 vs. 15	0.75 (0.64, 0.89)	0.94 (0.71, 1.25)	0.79 (0.66, 0.94)	0.74 (0.62, 0.88)	0.83 (0.69, 1.01)	0.8 (0.66, 0.97)	0.77 (0.55, 1.07)
	35 vs. 25	0.83 (0.75, 0.92)	0.82 (0.69, 0.97)	0.83 (0.74, 0.92)	0.82 (0.74, 0.91)	0.73 (0.65, 0.83)	0.74 (0.66, 0.84)	0.78 (0.65, 0.93)
	45 vs. 35	0.77 (0.67, 0.87)	0.78 (0.63, 0.97)	0.72 (0.63, 0.83)	0.75 (0.66, 0.86)	0.69 (0.59, 0.81)	0.68 (0.58, 0.81)	0.68 (0.53, 0.88)
Antenatal Visits								
	4 vs. 0	1.35 (1.23, 1.49)	1.47 (1.24, 1.75)	1.38 (1.25, 1.54)	1.22 (1.1, 1.36)	1.3 (1.14, 1.48)	1.12 (1, 1.27)	1.5 (1.28, 1.77)
	8 vs. 4	1.22 (1.09, 1.37)	1.14 (0.94, 1.38)	1.19 (1.06, 1.33)	1.21 (1.07, 1.36)	1.25 (1.07, 1.46)	1.26 (1.09, 1.45)	1.18 (0.98, 1.42)
Previous Modern Contraceptive Use								
	Yes vs. No	1.14 (1.06, 1.21)	1.07 (0.95, 1.2)	1.12 (1.04, 1.2)	1.11 (1.03, 1.19)	1.08 (0.98, 1.19)	1.07 (0.97, 1.18)	1.05 (0.93, 1.19)
Delivery Year								
	2004 vs. 1993	4.7 (4.18, 5.29)	8.95 (7.17,11.19)	5.18 (4.56, 5.88)	4.87 (4.33, 5.48)	5.18 (4.5, 5.95)	5.2 (4.49, 6.03)	—
	2018 vs. 2004	4.77 (3.87, 5.88)	4.3 (3.09, 5.99)	4.75 (3.84, 5.88)	4.98 (4.02, 6.17)	5.87 (4.32, 7.97)	5.92 (4.33, 8.1)	3.23 (2.43, 4.3)
Years of Education								
	7 vs. 0	1.63 (1.28, 2.08)	1.69 (1.16, 2.44)	1.63 (1.27, 2.1)	1.28 (1.04, 1.58)	1.12 (0.84, 1.49)	1.11 (0.83, 1.5)	1.51 (1.04, 2.19)
	12 vs. 7	1.51 (1.21, 1.88)	1.51 (1.09, 2.09)	1.47 (1.17, 1.85)	1.58 (1.28, 1.97)	1.63 (1.21, 2.19)	1.69 (1.27, 2.25)	1.39 (0.99, 1.95)
Previous Pregnancies								
	+1	0.8 (0.77, 0.83)	0.79 (0.74, 0.84)	0.8 (0.77, 0.83)	0.79 (0.76, 0.82)	—	—	0.78 (0.74, 0.83)
Pregnancy Intention								
	Yes vs. No	1.23 (1.14, 1.31)	1.26 (1.12, 1.41)	1.23 (1.14, 1.32)	1.25 (1.17, 1.35)	1.18 (1.08, 1.29)	1.21 (1.1, 1.33)	1.2 (1.06, 1.35)
	Other vs. No	1.13 (0.56, 2.31)	0.46 (0.12, 1.74)	1.19 (0.6, 2.37)	1.15 (0.57, 2.32)	0.86 (0.4, 1.85)	0.88 (0.4, 1.96)	0.96 (0.22, 4.23)
Nationality vs. South African								
	Mozambican	0.42 (0.39, 0.45)	0.45 (0.4, 0.5)	0.42 (0.39, 0.45)	0.39 (0.37, 0.42)	0.33 (0.3, 0.36)	0.31 (0.29, 0.34)	0.7 (0.62, 0.79)
	Other	0.1 (0.06, 0.17)	0.14 (0.06, 0.32)	0.1 (0.06, 0.17)	0.09 (0.05, 0.15)	0.08 (0.05, 0.15)	0.07 (0.04, 0.13)	0.09 (0.05, 0.15)
Residency Status vs. Permanent								
	Temporary	1.91 (1.66, 2.2)	1.75 (1.36, 2.27)	1.85 (1.59, 2.15)	1.84 (1.59, 2.13)	1.74 (1.45, 2.09)	1.69 (1.4, 2.03)	1.87 (1.47, 2.38)
	Other	1.96 (1.55, 2.46)	1.91 (1.31, 2.79)	1.97 (1.55, 2.51)	1.99 (1.57, 2.52)	1.43 (1.08, 1.89)	1.43 (1.07, 1.91)	2.08 (1.48, 2.93)
Student								
	Yes vs. No	1.91 (1.72, 2.12)	2.14 (1.8, 2.55)	2.01 (1.8, 2.24)	1.92 (1.72, 2.14)	1.86 (1.65, 2.1)	1.9 (1.68, 2.15)	1.55 (1.26, 1.9)
Absolute household socioeconomic status								
	+1 increase in socioeconomic status index	—	—	—	—	—	—	1.92 (1.67, 2.22)

eTable 10: Sensitivity Analyses for Health Facility Delivery Model

		Primary Analysis	Births < 6 months before observation date	Births < 2 years before observation date	Full singleton births	First recorded births	Full singleton first recorded births	Absolute household socioeconomic status available
n		50,956	20,275	47,787	49,756	35,749	34,960	25,711
Age								
	25 vs. 15	0.74 (0.63, 0.86)	0.88 (0.67, 1.14)	0.78 (0.66, 0.92)	0.72 (0.61, 0.85)	0.82 (0.69, 0.98)	0.78 (0.65, 0.94)	0.73 (0.53, 1)
	35 vs. 25	0.81 (0.74, 0.89)	0.84 (0.72, 0.99)	0.79 (0.71, 0.87)	0.81 (0.74, 0.89)	0.73 (0.65, 0.81)	0.75 (0.67, 0.84)	0.78 (0.66, 0.93)
	45 vs. 35	0.81 (0.72, 0.92)	0.82 (0.67, 1)	0.77 (0.68, 0.88)	0.8 (0.71, 0.91)	0.75 (0.64, 0.87)	0.74 (0.63, 0.86)	0.72 (0.57, 0.92)
Antenatal Visits								
	4 vs. 0	1.13 (1.01, 1.27)	1.02 (0.86, 1.22)	1.1 (0.98, 1.24)	1.15 (1.03, 1.29)	1.18 (0.99, 1.41)	1.19 (1.02, 1.38)	1.48 (1.27, 1.72)
	8 vs. 4	1.3 (1.19, 1.43)	1.35 (1.15, 1.58)	1.33 (1.2, 1.48)	1.18 (1.06, 1.3)	1.25 (1.12, 1.41)	1.13 (1, 1.29)	1.07 (0.9, 1.27)
Previous Modern Contraceptive Use								
	Yes vs. No	1.17 (1.1, 1.25)	1.12 (1.01, 1.25)	1.15 (1.07, 1.24)	1.14 (1.06, 1.23)	1.12 (1.02, 1.22)	1.11 (1.02, 1.22)	1.01 (0.9, 1.14)
Delivery Year								
	2004 vs. 1993	2.77 (2.49, 3.07)	3.2 (2.67, 3.83)	2.9 (2.6, 3.23)	2.82 (2.53, 3.14)	2.74 (2.44, 3.09)	2.78 (2.44, 3.16)	—
	2018 vs. 2004	4.05 (3.39, 4.84)	4.38 (3.3, 5.81)	4.36 (3.63, 5.24)	4.05 (3.37, 4.87)	4.71 (3.69, 6.01)	4.54 (3.54, 5.83)	2.3 (1.81, 2.93)
Years of Education								
	7 vs. 0	1.67 (1.34, 2.08)	1.64 (1.17, 2.3)	1.21 (0.99, 1.48)	1.55 (1.24, 1.95)	1.1 (0.84, 1.44)	1.31 (0.98, 1.74)	1.57 (1.1, 2.24)
	12 vs. 7	1.32 (1.07, 1.62)	1.42 (1.05, 1.91)	1.39 (1.13, 1.7)	1.43 (1.16, 1.76)	1.44 (1.09, 1.9)	1.55 (1.19, 2)	1.25 (0.9, 1.74)
Previous Pregnancies								
	+1	0.82 (0.79, 0.85)	0.8 (0.76, 0.85)	0.81 (0.78, 0.84)	0.81 (0.78, 0.84)	—	—	0.81 (0.76, 0.85)
Pregnancy Intention								
	Yes vs. No	1.15 (1.08, 1.23)	1.13 (1.02, 1.26)	1.14 (1.07, 1.22)	1.17 (1.09, 1.24)	1.12 (1.02, 1.22)	1.14 (1.05, 1.24)	1.11 (0.99, 1.24)
	Other vs. No	1.47 (0.86, 2.51)	1.38 (0.57, 3.36)	1.43 (0.82, 2.51)	1.47 (0.86, 2.52)	1.21 (0.68, 2.15)	1.23 (0.7, 2.16)	0.71 (0.21, 2.43)
Nationality vs. South African								
	Mozambican	0.43 (0.41, 0.46)	0.46 (0.41, 0.51)	0.42 (0.4, 0.45)	0.42 (0.4, 0.45)	0.36 (0.33, 0.39)	0.35 (0.32, 0.38)	0.73 (0.65, 0.82)
	Other	0.14 (0.08, 0.22)	0.19 (0.08, 0.43)	0.13 (0.08, 0.21)	0.12 (0.07, 0.2)	0.12 (0.07, 0.2)	0.1 (0.06, 0.18)	0.11 (0.07, 0.19)
Residency Status vs. Permanent								
	Temporary	1.4 (1.24, 1.58)	1.29 (1.04, 1.59)	1.38 (1.21, 1.56)	1.32 (1.17, 1.49)	1.26 (1.08, 1.48)	1.17 (1.01, 1.37)	1.2 (1, 1.45)
	Other	1.47 (1.21, 1.77)	1.19 (0.88, 1.6)	1.45 (1.19, 1.76)	1.44 (1.18, 1.76)	1.08 (0.85, 1.36)	1.02 (0.8, 1.29)	1.4 (1.07, 1.84)
Student								
	Yes vs. No	1.81 (1.64, 1.99)	2.06 (1.75, 2.42)	1.85 (1.67, 2.05)	1.86 (1.68, 2.06)	1.76 (1.57, 1.97)	1.87 (1.66, 2.1)	1.36 (1.13, 1.65)
Absolute household socioeconomic status								
	+1 increase in socioeconomic status index	—	—	—	—	—	—	1.63 (1.42, 1.86)

Supplemental Figures

eFigure 1. Other descriptive variables by delivery year

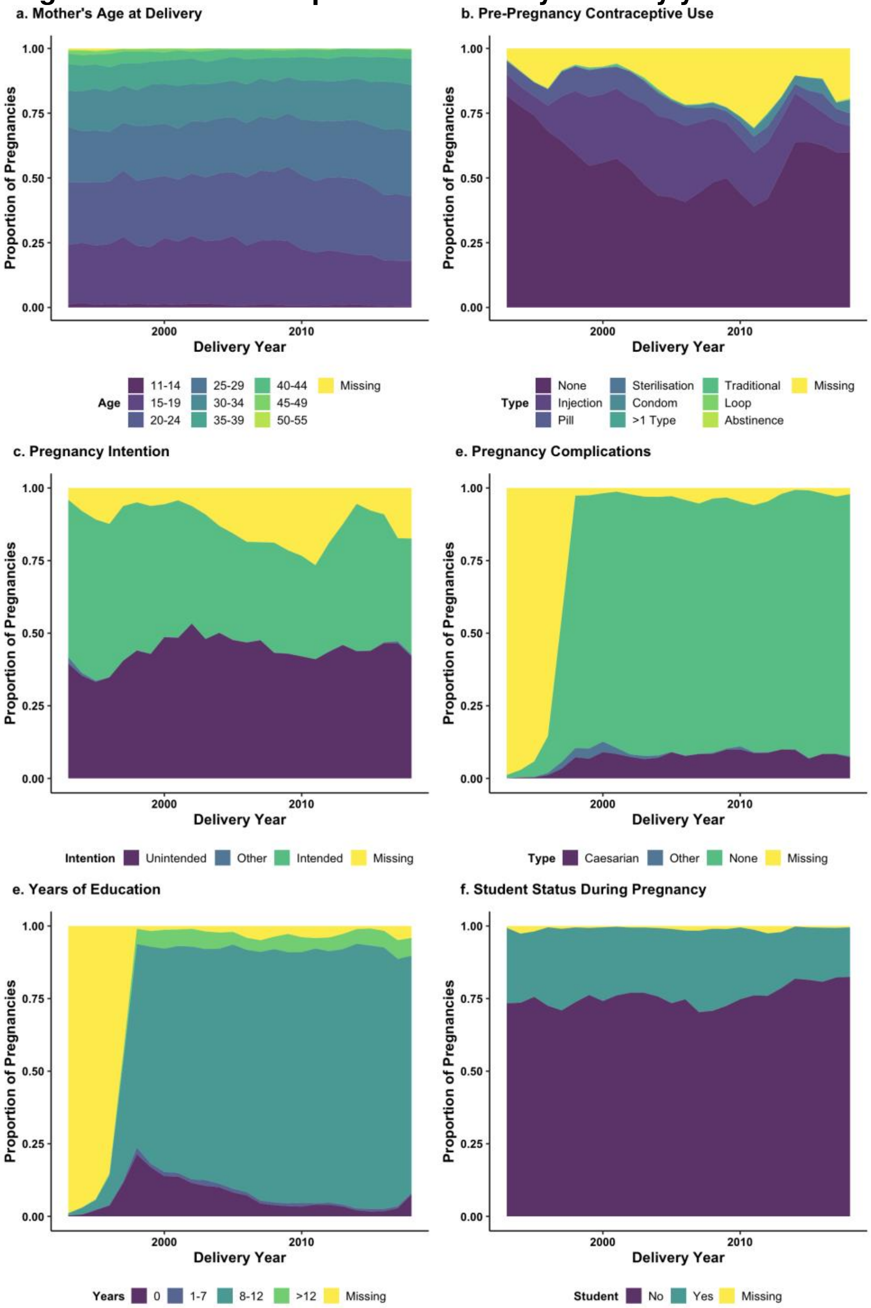

All subfigures present the proportion of pregnancies per year. eFigure 1a shows maternal age by delivery year, with ages categorized into ten bins: 11-14, 15-19, 20-24, 25-29, 30-34, 35-39, 40-44, 45-49, 50-55, and missing. eFigure 1b shows pre-pregnancy contraceptive use by delivery year. eFigure 1c shows pregnancy intention by delivery year. eFigure 1d shows pregnancy complications use by delivery year. eFigure 1e shows the reported years of education by delivery year, with South African and Mozambican school, as well as adult educational programs such as the adult basic education and training and national qualification framework in South Africa to their equivalent years of education. eFigure 1f shows student status by delivery year.

eFigure 2. Proportional odds assumption for ordinal antenatal visits model

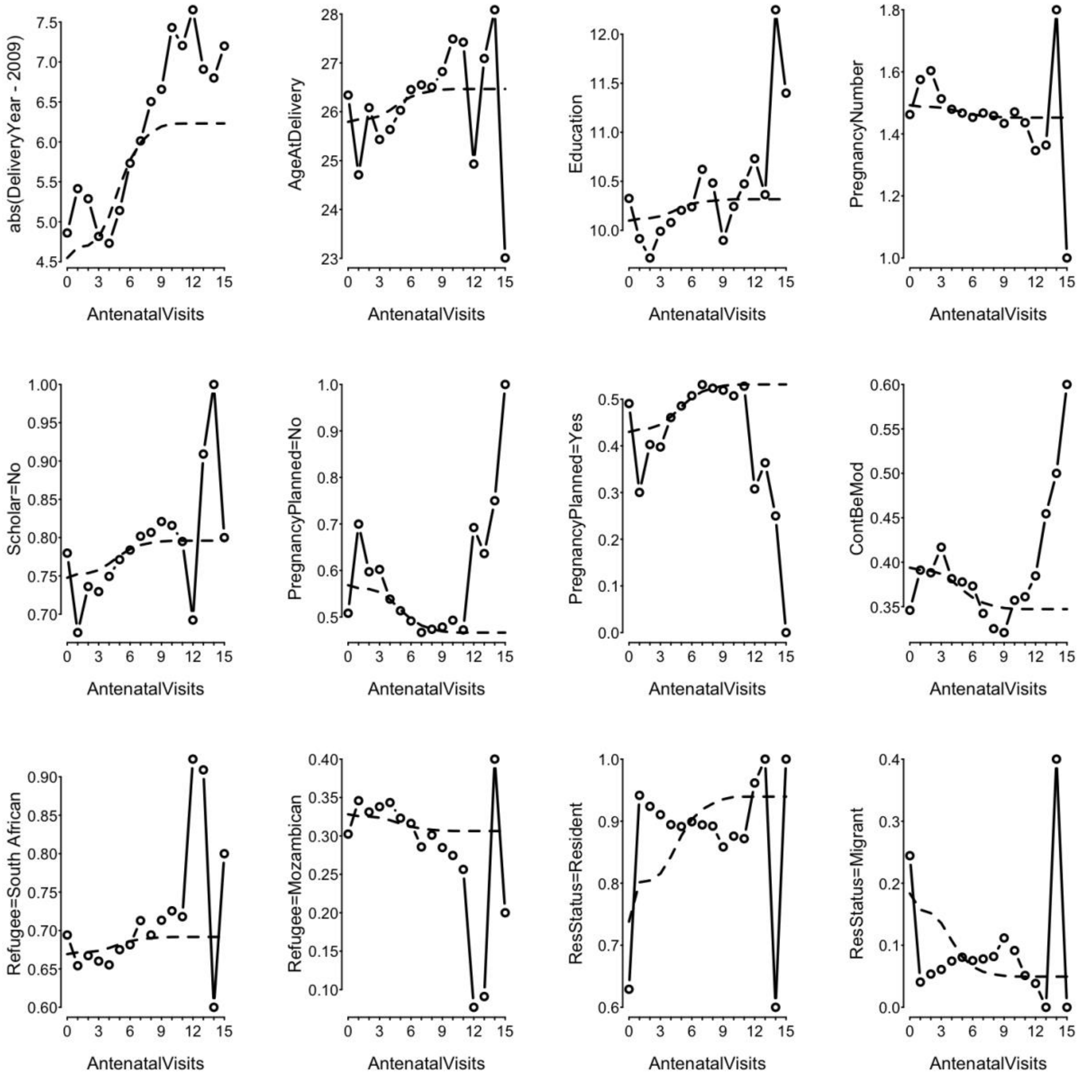

eFigure 2 shows the ordinality of Y (antenatal visits) for each predictor. It assesses how varying the number of antenatal visits relates to the mean of each predictor and the trend of the relationship. The solid lines represent the simple stratified means, whereas the dashed lines represent the expected value of the predictor given antenatal visits assuming the proportional odds assumption holds.[3]

eFigure 3. Partial effects plot by delivery year

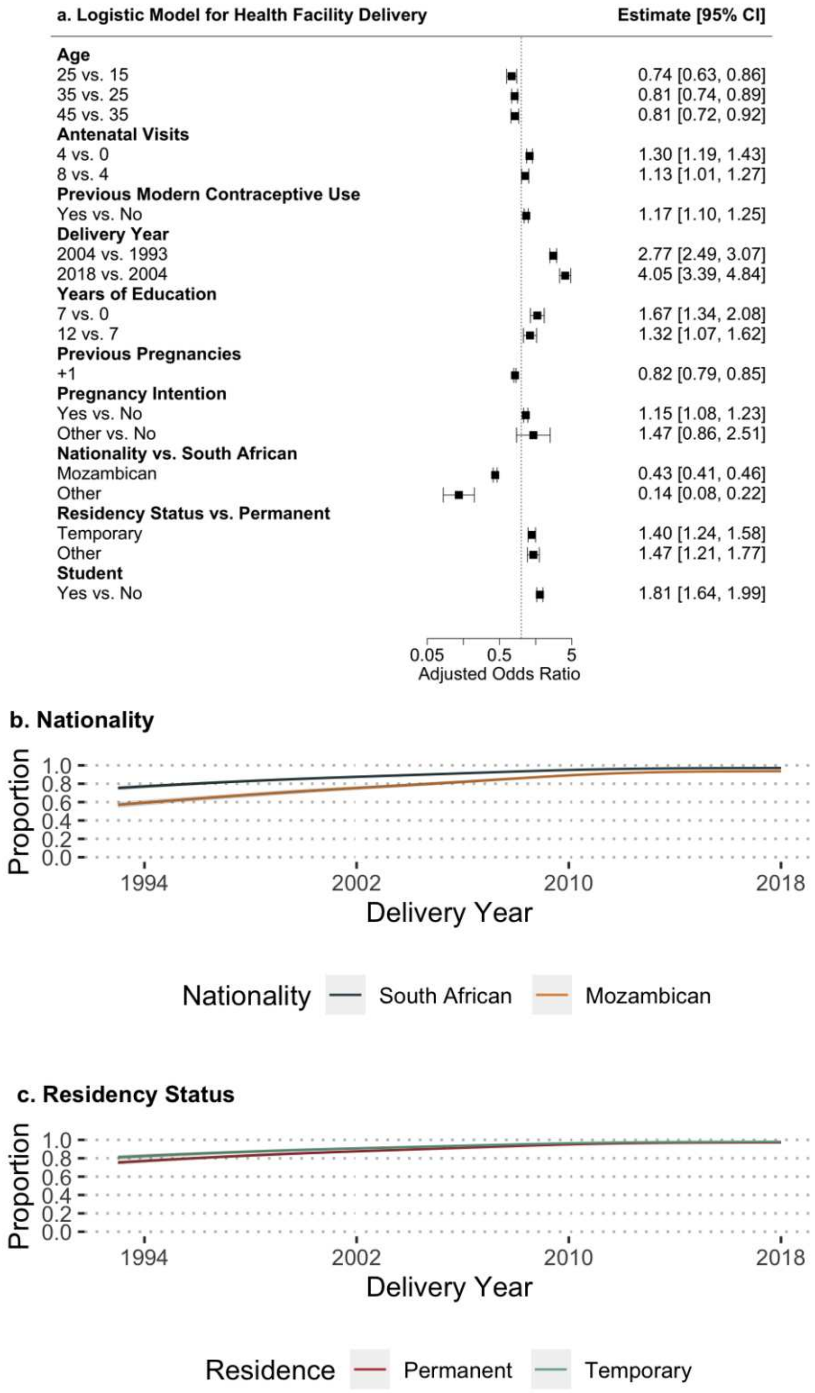

eFigure 3 shows the forest plots for each covariate in (a) the logistic model for skilled birth attendant presence. In (a), we present the adjusted odds ratio (Estimate) and the 95% Confidence Interval for each covariate. In (b) and (c), we present the proportion of deliveries at a health facility over time, adjusted for age at delivery, number of antenatal visits, previous modern contraceptive use, years of education, number of previous pregnancies, pregnancy intention, and student status. Mothers of Mozambican descent were less likely to report delivering a health facility for the duration of the study period as compared to South African mothers (b), although this difference decreased over time. Mothers who identified as temporary migrants, however, were more likely than mothers who identified as permanent migrants to deliver in a health facility (c). Note that in the absence of an interaction term, the lines are forced to be parallel. We excluded “Other” from this figure like in Figure 4 because of the very small group of deliveries among those individuals.

eFigure 4. Logistic model calibration

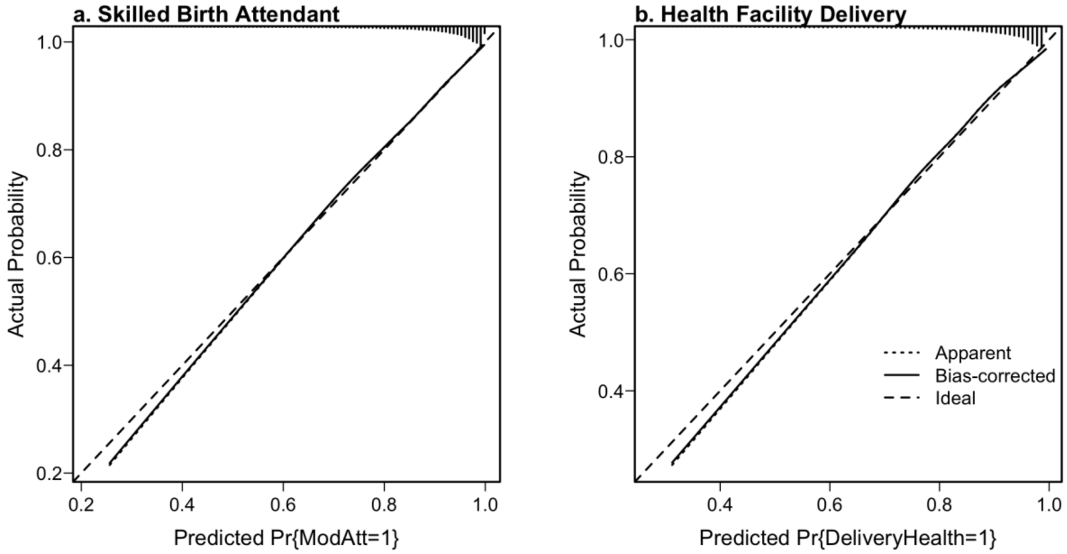

eFigure 4 shows the calibration curves for the a) skilled birth attendant and b) health facility delivery logistic models. The x-axis shows the predicted probability of skilled birth attendance (a) and health facility delivery (b) in each respective model, while the y-axis shows the actual probability. The dashed line on both graphs shows the ideal calibration curve, the dotted line shows the apparent calibration curve, and the solid line shows the bias-corrected calibration curve after 1000 bootstrapped repetitions. The tick marks at the top show the distribution of the predicted risks. The mean squared error, based on 48,646 participants, is 0.003 for the skilled birth attendant model (a) and, based on 50,956 participants, 0.006 for the health facility delivery model. These plots were created via the “rms” package in R.[4,5]

eFigure 5. Predictive model sensitivity analysis forest plots

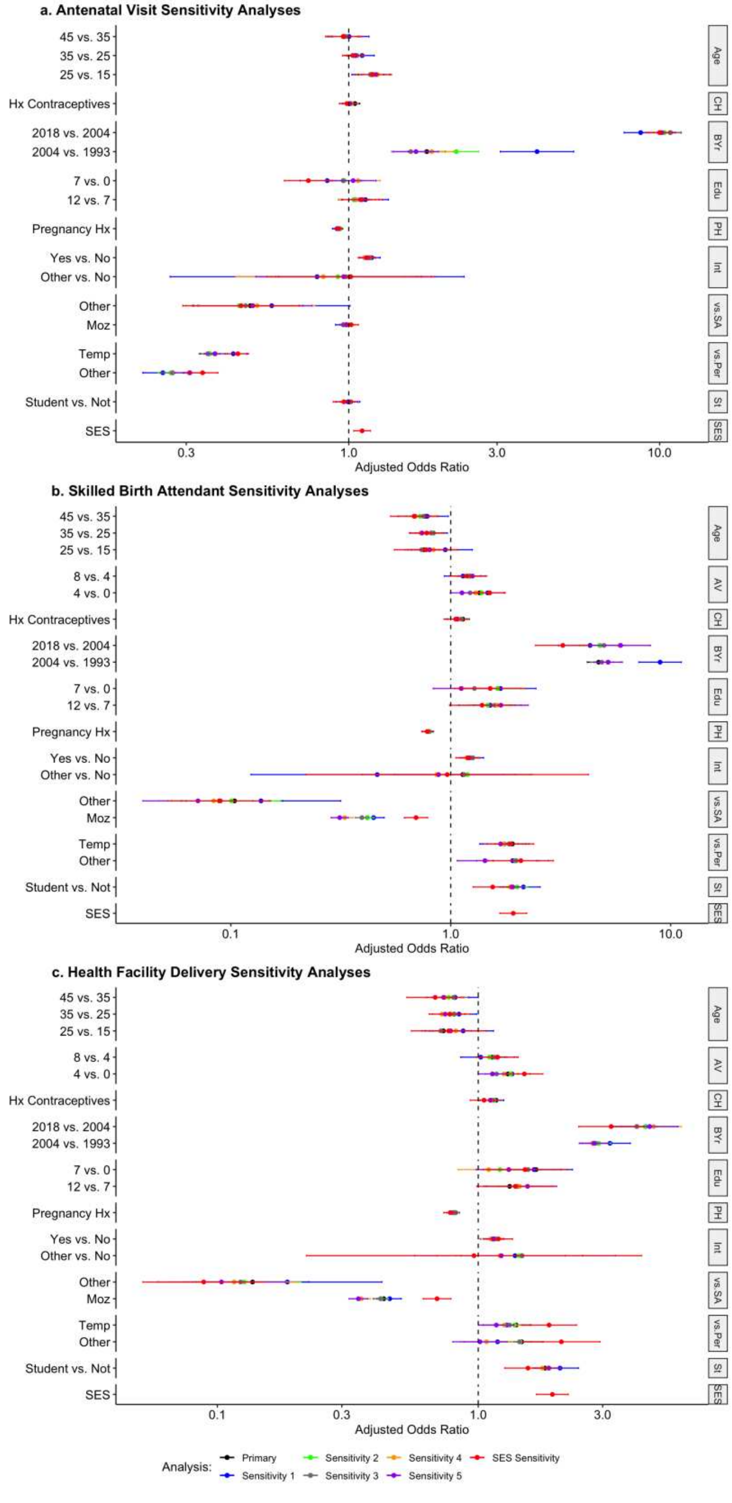

eFigure 5 shows forest plots for each covariate in (a) the ordinal model for antenatal visit attendance, (b) the logistic model for skilled birth attendant presence, and (c) the logistic model for health facility delivery and the corresponding sensitivity analyses. All three plots present the adjusted odds ratio (Estimate) and the 95% Confidence Interval for each covariate. The primary analysis (black) is the same as Figure 3. Sensitivity analysis 1 (blue) includes births that occurred 6 or fewer months before the observation date. Sensitivity analysis 2 (green) includes births that occurred 2 or fewer years before the observation date. Sensitivity analysis 3 (grey) includes only full-term singleton births. Sensitivity analysis 4 (orange) includes only first recorded births. Sensitivity analysis 5 (purple) includes only full-term singleton first recorded births. SES Sensitivity analysis (red) includes only deliveries in years where socioeconomic status data were collected in pregnant women (2001, 2003, 2005, 2007, 2009, 2011, 2013, 2014, 2015, 2016, 2017, 2018). Each plot is faceted by covariates of interest: Age, AV (antenatal visits), CH (contraceptive history), BYr (delivery year), Edu (years of education), PH (pregnancy history), Int (pregnancy intention), vs. SA (nationality versus South African), vs. Per (residency status versus permanent), St (student status), and SES (socioeconomic status – an absolute index of household assets)[2].

References

1. Hubacher D, Trussell J. A definition of modern contraceptive methods. *Contraception*. 2015;92(5):420-421. doi:10.1016/j.contraception.2015.08.008
2. Kabudula CW, Houle B, Collinson MA, Kahn K, Tollman S, Clark S. Assessing Changes in Household Socioeconomic Status in Rural South Africa, 2001–2013: A Distributional Analysis Using Household Asset Indicators. *Soc Indic Res*. 2017;133(3):1047-1073. doi:10.1007/s11205-016-1397-z
3. Harrell FE. *Regression Modeling Strategies*. Springer-Verlag; 2015.
4. Harrell Jr FE. *Rms: Regression Modeling Strategies.*; 2020. Accessed December 7, 2020. https://CRAN.R-project.org/package=rms
5. Team RC. *R: A Language and Environment for Statistical Computing*. R Foundation for Statistical Computing; 2019. https://www.R-project.org/
